# Oral formulation of DPP-4 inhibitor plus Quercetin improves metabolic homeostasis in type 1 diabetic rats

**DOI:** 10.1038/s41598-018-33727-x

**Published:** 2018-10-17

**Authors:** Pedro Henrique de A. Miranda, Kissyla Christine Duarte Lacerda, Carolina Morais Araújo, José Mario Barichello, Wanderson Geraldo Lima, Daniela Caldeira Costa

**Affiliations:** 1 Laboratório de Bioquímica Metabólica (LBM), Departamento de Ciências Biológicas (DECBI), Universidade Federal de Ouro Preto (UFOP), Ouro Preto, MG 35400-000 Brazil; 2 Programa de Pós-Graduação em Ciências Biológicas, Universidade Federal de Ouro Preto (UFOP), Ouro Preto, MG 35400-000 Brazil; 3 Programa de Pós-Graduação em Saúde e Nutrição, Universidade Federal de Ouro Preto (UFOP), Ouro Preto, MG 35400-000 Brazil; 4 Laboratório de Morfopatologia, Departamento de Ciências Biológicas (DECBI), Universidade Federal de Ouro Preto (UFOP), Ouro Preto, MG 35400-000 Brazil; 5 Escola de Farmácia, Universidade Federal de Ouro Preto (UFOP), Ouro Preto, MG 35400-000 Brazil; 6 Laboratório de Tecnologia Farmacêutica, Centro de Ciências Químicas, Farmacêutica e de Alimento, Universidade Federal de Pelotas (UFPEL), Rio Grande do Sul, RS 96160-990 Brazil

## Abstract

This study aimed to investigate the potential of an oral formulation (QV formulation) containing Quercetin and a Dipeptidyl Peptidase-4 Inhibitor (DPP-4 inhibitor), Vildagliptin, in improving metabolic homeostasis in type 1 diabetes model. Female albino Fischer rats were divided into four groups: untreated control animals (C), untreated diabetic animals (D), diabetic animals treated with QV formulation (DQV), and diabetic animals treated with insulin (DI). Diabetes was induced by injection of alloxan (135 mg kg body mass)^−1^ and confirmed by glycemic test. After the 30-day treatment period, biochemical parameters were analyzed in the pancreas, liver, and serum. Histopathological changes in pancreatic tissue were examined by Hematoxyline & Eosin staining and the insulin content in the islet measured by immunohistochemistry with anti-insulin antibody. The glycogen content in the hepatocytes was quantified by Periodic Schiff Acid staining. The QV formulation reduced the glycemia, preserved the pancreatic architecture, increased insulin levels, furthermore ameliorated lipid profile and to promote higher survival rate of animals. Together, our data suggest that the QV formulation treatment was able to normalize metabolic homeostasis in type 1 diabetic rats.

## Introduction

Type 1 diabetes (T1DM), prevalent in 5 to 10% of the diabetic population, is a result of the immune-mediated destruction of pancreatic beta cells. It manifests earlier than type 2 diabetes (T2DM), and the incidence among children has been increasing in many countries, particularly in individuals under 15 years old^1^. The individuals affected may find it difficult to perform glycemic control, resulting in less adherence to treatment. This is mainly due to invasive treatment, which consists of the subcutaneous administration of insulin at regular intervals. Conventional treatment generates pain, anxiety, and loss of quality of life, which impacts the life of individuals and their families. In addition, the high financial cost results in medical care and decreased productivity, making this disease burdensome^2^. Different strategies have been developed to provide greater comfort for patients and to prevent frequent episodes of severe hypoglycemia, such as glucose sensors, oral and dermal insulins^3^, and oral spray. However, access to these innovations is unfeasible for a large part of the population due to the high cost, considering that 75% of patients with T1DM live in low-income countries. Recently our group demonstrated that drugs indicated for treatment of T2DM have been studied in T1DM and show a variable degree of benefits^4^. DPP-4 inhibitors participate in numerous biological processes, via glycemic control, weight reduction, and improvement in beta cell function and cardiovascular risk markers^5^, resulting in additional beneficial effects in metabolic homeostasis. In addition, in T1DM, DPP-4 inhibitors as add-on to insulin therapy can reduce the daily insulin requirements^6^. Animal studies indicate that Vildagliptin, an oral DPP-4 inhibitor, has a promising effect on T1DM, stimulating the neogenesis of pancreatic beta cells^7^. Other authors have also reported effects of Vildagliptin on the stimulation of insulin secretion^8^ and on the maintenance and repair of beta cells^9^ in animal model. However, no studies to date have demonstrated the effect of Vildagliptin on the regulation of metabolism in the T1DM model. In addition, the use of Quercetin, a flavonoid present in several foods of plant origin, has also been demonstrated to have antidiabetic potential, improving the hyperglycemic state, reducing the incidence of peripheral complications^10^ and increasing the secretion of insulin and protection of pancreatic cells^11^. Studies have shown that both Quercetin and Vildagliptin, when administrated alone, have antidiabetic potential, but their combined effect on metabolic homeostasis in the T1DM model is controversial. Considering different mechanisms of action, either by direct effects of Vildagliptin on the inhibition of DPP-4 enzyme, or by the antioxidant activity of Quercetin, we hypothesized that the combination of these two compounds are capable of to generate benefits in biochemical and histological parameters, promoting an improvement in metabolic homeostasis in type 1 diabetic rats.

## Results

**Figure 1:**
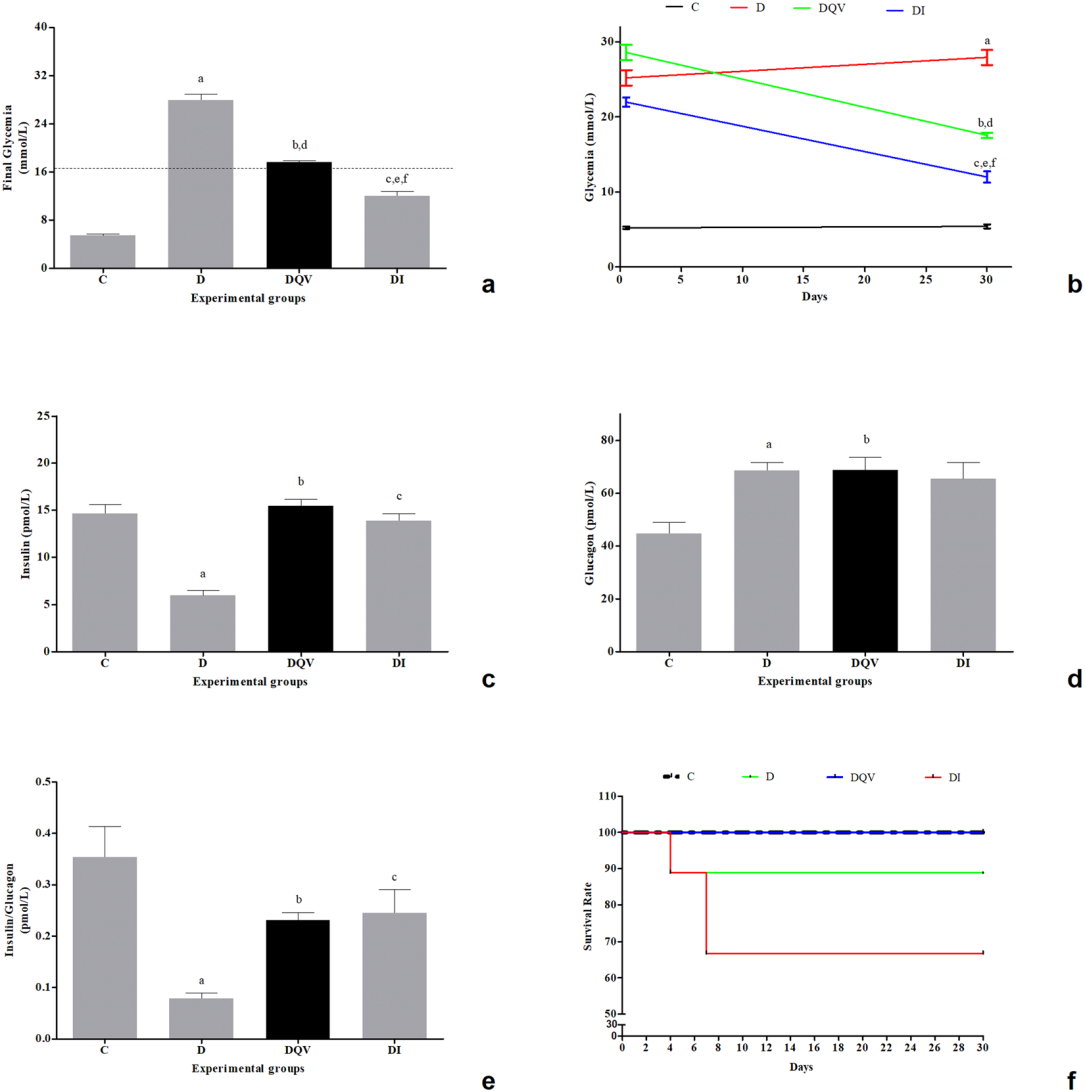
QV formulation reduces glycemia, maintains a positive insulin/glucagon ratio and promote higher survival rate of experimental groups over 30 days. (**a**) Final glycemia of treated or untreated animals, with QV formulation or insulin. Dashed line shows threshold for animals to be considered diabetic or not (blood glucose concentration ≥16 mmol/L). a: C vs D (p < 0.0001); b: C vs DQV (p < 0.0001); c: C vs DI (p < 0.0001); d: D vs DQV (p < 0.0001); e: D vs DI (p < 0.0001); f: DQV vs DI (p < 0.0001). (**b**) Fasting blood glucose × Time. (**c**) Serum insulin levels. a: C vs D (p < 0.0001); b: D vs DQV (p < 0.0001); c: D vs DI (p < 0.0001). (**d**) Serum glucagon levels. a: C vs D (p = 0.0427); b: C vs DQV (p = 0.0155). (**e**) Insulin/Glucagon ratio. a: C vs D (p < 0.0001); b: D vs DQV (p = 0.0143); c: D vs DI (p = 0.0255). (**f**) Survival rate. (Black dashed line indicates group C. Green colored line indicates group D. Blue colored line indicates DQV group. Red colored line indicates group DI). C, control group (n = 6); D, diabetic group (n = 10); DQV, diabetic group treated with QV formulation (n = 14); DI, diabetic group treated with insulin (n = 10). Results expressed as mean ± standard error.

After 30 days of treatment, the group receiving insulin alone and the group that received the QV formulation had a reduction in the hyperglycemic condition (Fig. 1a). The percentage of blood glucose reduction was 70% in the animals that received the QV formulation, compared to the approximately 55% glycemic reduction in animals receiving insulin alone (Fig. 1b). The serum analysis revealed that QV formulation increased insulin levels to values similar to the control and insulin groups (Fig. 1c), but did not change glucagon levels (Fig. 1d), maintaining a positive insulin/glucagon ratio (Fig. 1e). In the diabetic group that received the QV formulation, all animals survived over the entire period, and the same was observed for the animals in the non-diabetic control group. In contrast, 11.2% of the untreated diabetic animals died within 4 days after confirmation of diabetes, and 33.3% of those treated once daily with insulin died within 7 days (Fig. 1f).

**Figure 2:**
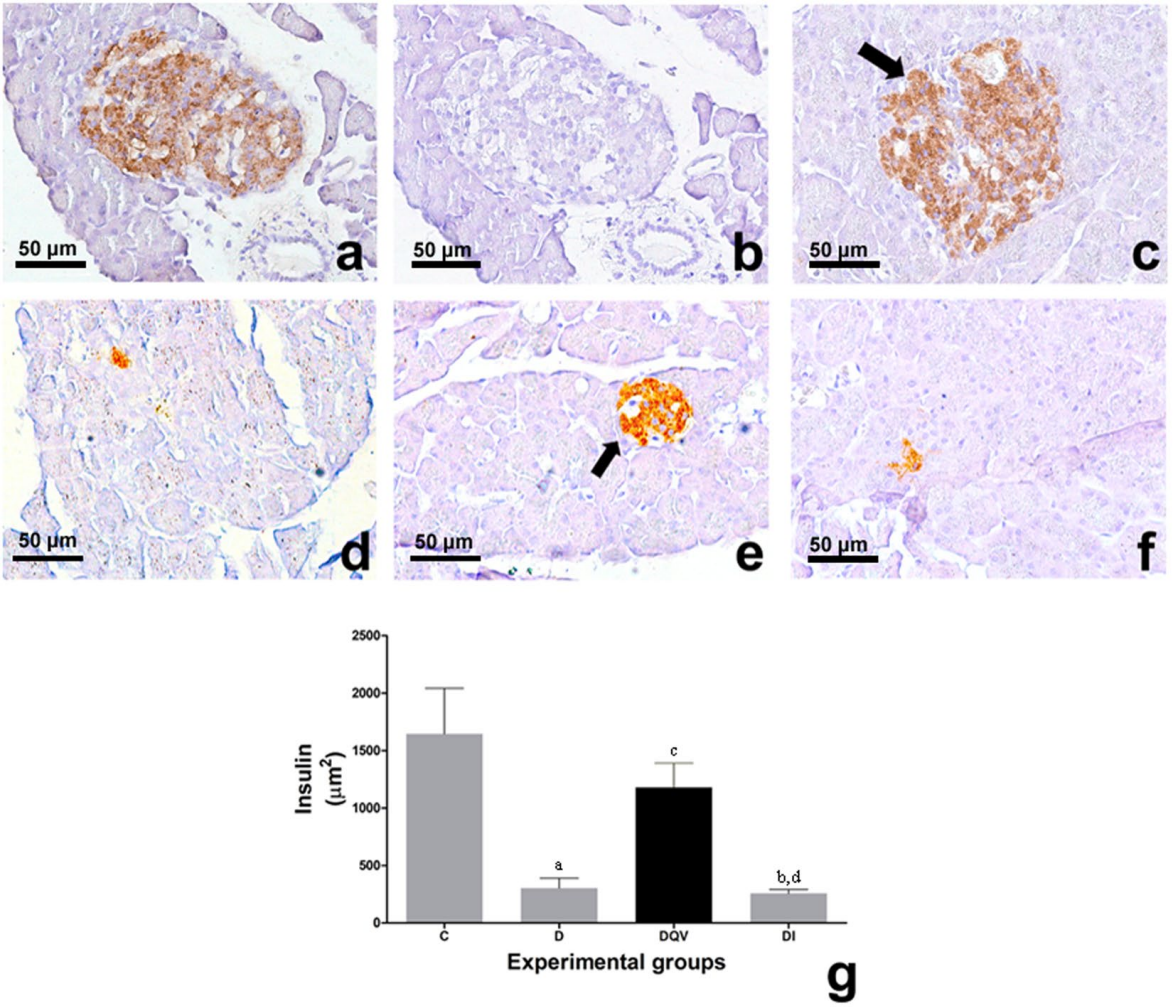
Treatment with QV formulation increased the biosynthesis of insulin in pancreatic islets. (**a**–**f**) The histological sections of the pancreas of experimental animals stained by immunohistochemistry in 440x magnification. (**a**,**b**) Positive and Negative control of coloration, respectively. (**c**) Control group showing the normal immunolabeling to insulin in pancreatic islets. (**d**) Diabetic group animals showing low immunolabeling to insulin. (**e**) Diabetic group animals treated with QV formulation with immunolabeling similar to control group. (**f**) Diabetic animals treated with insulin presenting immunolabeling similar to diabetic group. The arrows shows the immunolabeled pancreatic islets. (**g**) Concentration of insulin in pancreatic islets. a: C vs D (p = 0.0003); b: C vs DI (p = 0.0003); c: D vs DQV (p = 0.0146); d: DQV vs DI (p = 0.0120). C, control group (n = 5); D, diabetic group (n = 8); DQV, diabetic group treated with QV formulation (n = 6); DI, diabetic group treated with insulin (n = 7). Results expressed as mean ± standard error.

Histological sections of pancreatic islets from the experimental groups reveled positive labeling with anti-insulin antibody (Fig. 2a–f). The QV formulation was able to increase insulin production by pancreatic beta cells (Fig. 2g).

**Figure 3:**
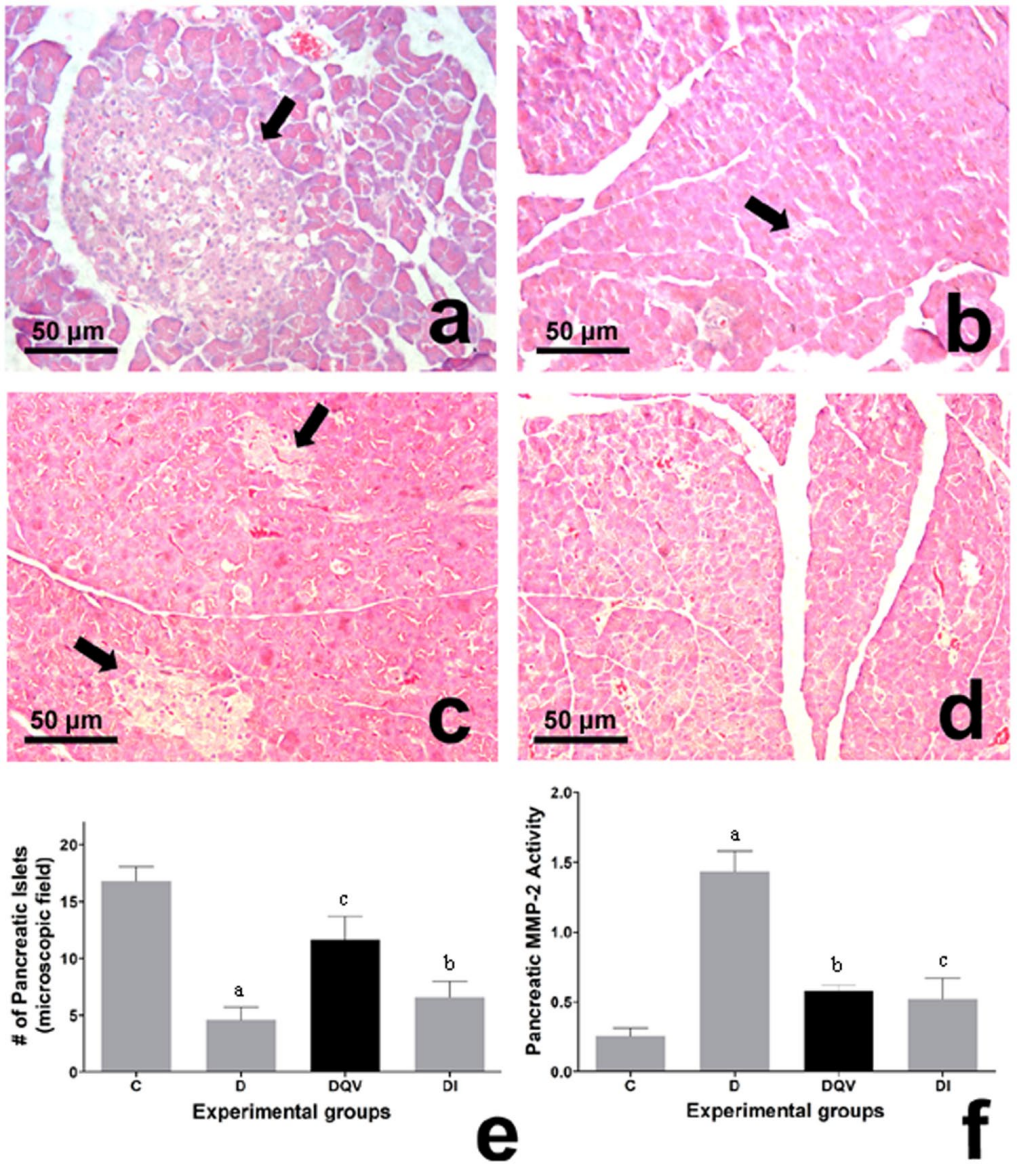
Treatment with QV formulation increases the number of pancreatic islets and reduces tissue architecture damage. (**a**–**d**) Histological sections of the pancreas of experimental animals stained by H&E in 440x magnification. (**a**–**d**) Normal architecture of pancreatic islets in groups. Arrows shows the pancreatic islets. (**e**) Number of pancreatic islets (p = 0.0002). a: C vs D (p = 0.0003); b: C vs DI (p = 0.0020); c: D vs DQV (p = 0.0289). (**f**) Pancreatic MMP-2 activity. a: C vs D (p = 0.0323); b: D vs DQV (p = 0.0447); c: D vs DI (p = 0.0379). C, control group (n = 5); D, diabetic group (n = 8); DQV, diabetic group treated with QV formulation (n = 6); DI, diabetic group treated with insulin (n = 7). Results expressed as mean ± standard error.

No inflammatory signs, degenerative or fibrotic areas were found in any of the experimental group animals, either in the acinar components or in the pancreatic islets (Fig. 3a–d). The number of pancreatic islets increased after administration of the QV formulation in the treated animals when compared to the untreated diabetic group, and remained similar in relation to the insulin-treated group (Fig. 3e). Evaluation of the remodeling capacity of the extracellular matrix promoted by the pancreatic MMP-2 (matrix metalloproteinase-2) revealed that treatments with the QV formulation or insulin reduced the MMP-2 activity in relation to the untreated diabetic group, and at levels similar to the control group (Fig. 3f).

**Figure 4:**
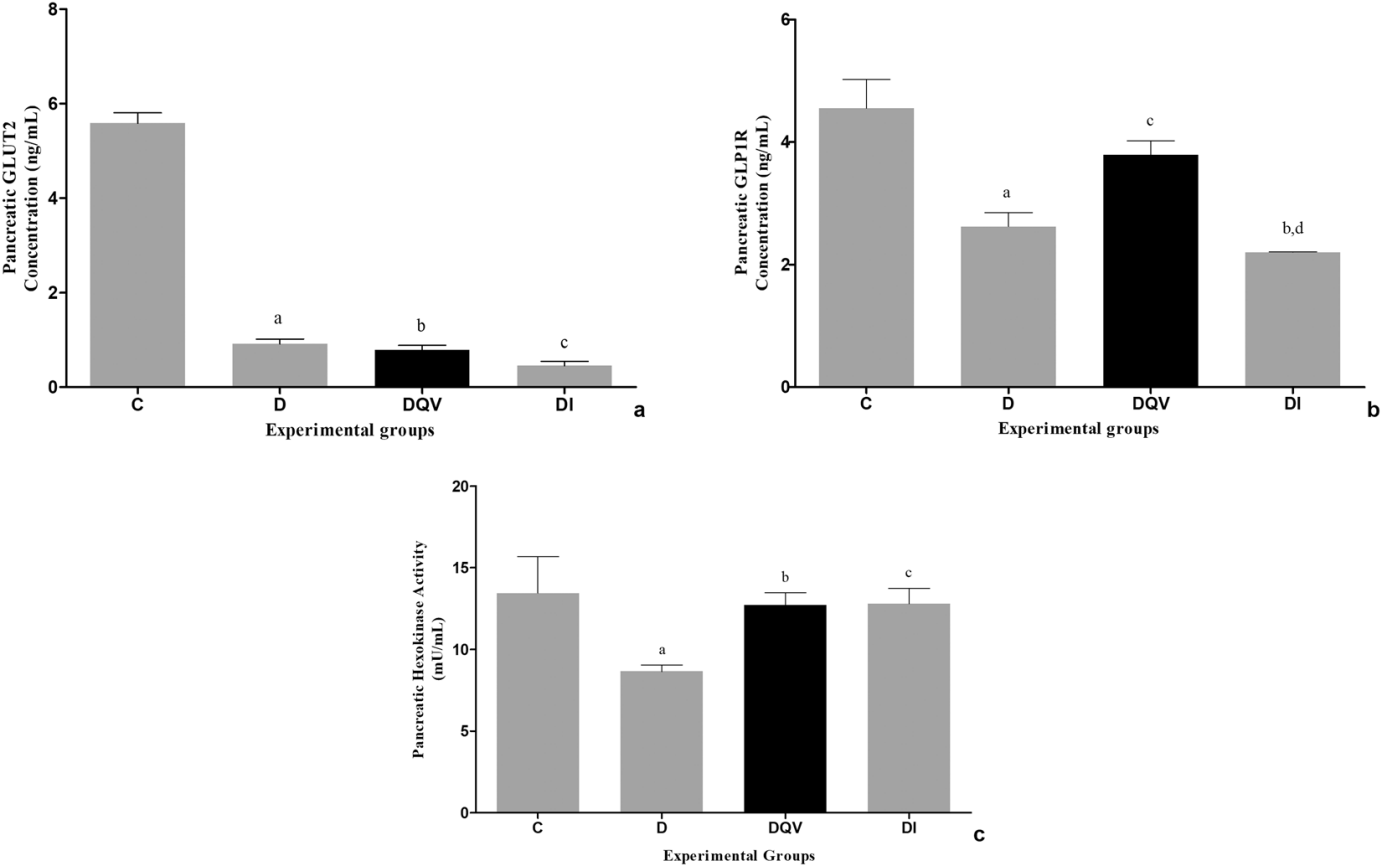
Treatment with QV formulation did not change the pancreatic GLUT2 levels, but increased both the GLP-1R concentration and activity of the hexokinase enzyme. (**a**) Concentration of pancreatic GLUT2. a: C vs D (p < 0.0001); b: C vs DQV (p < 0.0001); c: C vs DI (p < 0.0001). (**b**) Concentration of pancreatic GLP-1R. a: C vs D (p < 0.0010); b: C vs DI (p = 0.0001); c: D vs DQV (p = 0.0461); d: DQV vs DI (p = 0.0044). (**c**) Activity of pancreatic hexokinase. a: C vs D (p = 0.0323); b: D vs DQV (p = 0.0447); c: D vs DI (p = 0.0379). C, control group (n = 5); D, diabetic group (n = 6); DQV, diabetic group treated with QV formulation (n = 7); DI, diabetic group treated with insulin (n = 5). Results expressed as mean ± standard error.

None of the treatments showed an effect on the GLUT2 (glucose transporter 2) level present in the pancreas (Fig. 4a). The GLP-1R (glucagon-like peptide-1 receptor) level revealed that the QV formulation enabled an increase the concentration of this receptor (Fig. 4b). The activity of the enzyme pancreatic hexokinase revealed that administration of the QV formulation reestablished the functional capacity of hexokinase in phosphorylating the glucose present in the medium (Fig. 4c).

**Figure 5:**
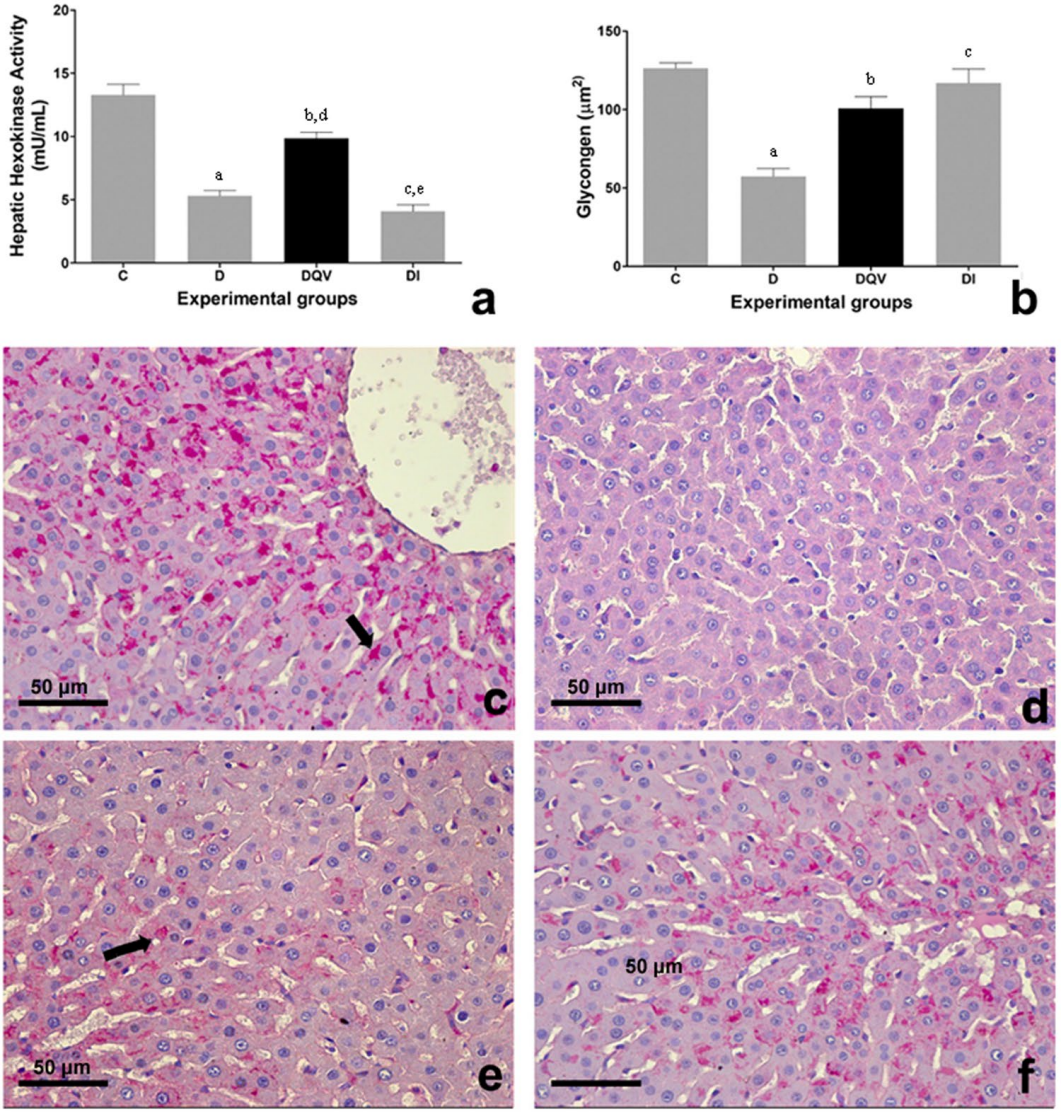
The activity of hepatic hexokinase and hepatic glycogen levels are increased by treatment with QV formulation. (**a**) Analysis of the enzymatic activity of hexokinase hepatic. a: C vs D (p < 0.0001); b: C vs DQV (p = 0.0017); c: C vs DI (p < 0.0001); d: D vs DQV (p < 0.0001); e: DQV x DI (p < 0.0001). C, control group (n = 5); D, diabetic group (n = 7); DQV, diabetic group treated with QV formulation (n = 13); DI, diabetic group treated with insulin (n = 7). (**b**) Concentration of glycogen. a: C vs D (p < 0.0001); b: D vs DQV (p = 0.0028); c: D vs DI (p = 0.0005). C, control group (n = 5); D, diabetic group (n = 5); DQV, diabetic group treated with QV formulation (n = 13); DI, diabetic group treated with insulin (n = 5). Results expressed as mean ± standard error. (**c**–**f**) Histological sections of the liver of experimental animals stained by PAS in 440x magnification. (**c**) Normal architecture of liver and glycogen deposition within the hepatocytes in control animals. (**d**) No glycogen deposition were seen in untreated diabetic animals. (**e**) Glycogen deposition in animals treated with QV formulation and (**f**) treated with insulin. Arrows showing glycogen deposition.

The activity of hexokinase in hepatocytes of animals that received QV formulation was reestablished (Fig. 5a). Both treatments increased the energetic reserve of glycogen in relation to the untreated diabetic group (Fig. 5b). The representation of hepatic tissues in photomicrography shows glycogen deposits stained by PAS (periodic schiff acid) (Fig. 5c–f).

**Figure 6:**
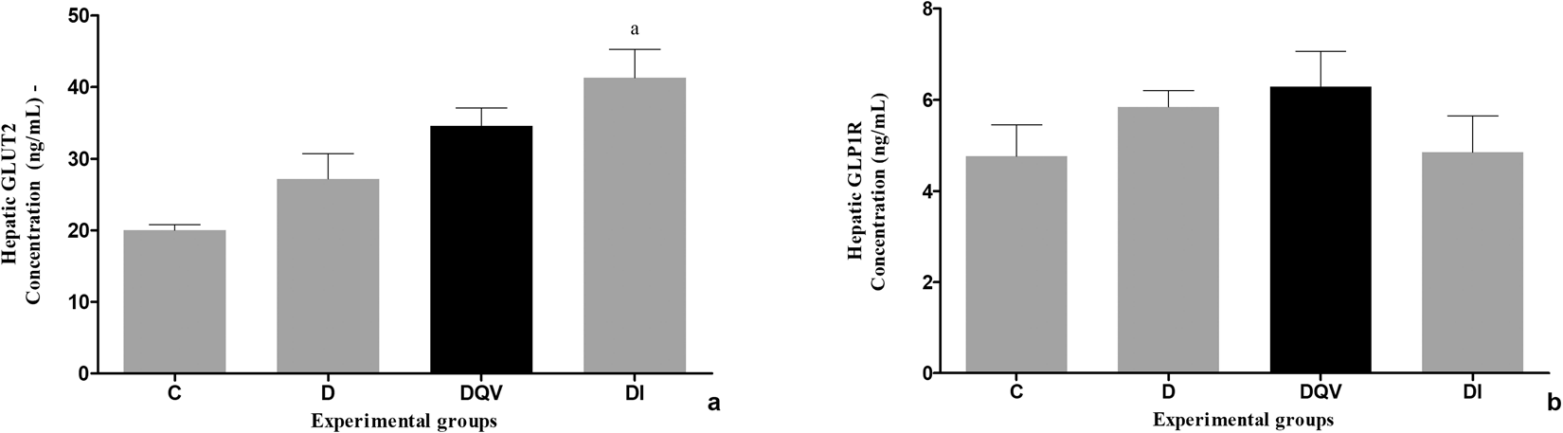
Treatment with QV formulation does not change the hepatic levels of GLUT2 and GLP-1R. (**a**) Concentration of hepatic GLUT2. a: C vs DI (p = 0.0054). (**b**) Concentration of hepatic GLP-1R (p = 0.3357). C, control group (n = 5); D, diabetic group (n = 6); DQV, diabetic group treated with QV formulation (n = 7); DI, diabetic group treated with insulin (n = 6). Results expressed as mean ± standard error.

GLUT2 concentration, as well as GLP-1R concentration present in the liver did not reveal differences between the experimental groups who received the treatments (Fig. 6a,b).

Lipid profile dosages showed that treatment with QV formulation reduced the levels of the non-HDL fraction (particle non high density lipoprotein-cholesterol - atherogenic particles) and increased HDL fraction in relation to the untreated diabetic group. No differences were found between groups in relation to cholesterol total, however the QV formulation exhibited lower triacylglycerol levels in relation to insulin administration. The QV formulation did not exhibit alterations in ALT (alanine aminotransferase), AST (aspartate aminotransferase) and albumin concentrations. Assessment of urea levels showed that treatment with QV formulation was able to promote an improvement in this parameter in comparison to the untreated diabetic group. In contrast, the serum creatinine levels showed no change in any of the groups evaluated (Table 1).

**Table 1:** Effect of QV formulation on lipid profile, renal and hepatic function caused by experimental diabetes.

Experimental Groups	C	D	DQV	DI
Non-HDL (mmol/L)	1.55 ± 0.19	2.24 ± 0.12^a^	1.78 ± 0.09^c^	2.18 ± 0.13^b^
HDL (mmol/L)	1.77 ± 0.12	0.73 ± 0.04^a^	1.03 ± 0.05^b,d^	0.88 ± 0.04^c^
Cholesterol total (mmol/L)	3.32 ± 0.21	3.00 ± 0.16	2.81 ± 0.07	3.06 ± 0.13
Triacylglycerol (mmol/L)	1.49 ± 0.04	2.63 ± 0.18^a^	2.05 ± 0.11	3.27 ± 0.30^b,c^
ALT (U/L)	17.02 ± 1.71	43.77 ± 3.85^a^	39.29 ± 1.13^b^	39.29 ± 2.18^c^
AST (U/L)	18.19 ± 0.94	40.16 ± 4.23^a^	36.84 ± 0.98^b^	53.84 ± 2.46^c,d,e^
Albumin (µmol/L)	583.2 ± 29.16	454.3 ± 7.7^a^	468.60 ± 5.2^b^	494.2 ± 15.51^c^
Urea (mmol/L)	7.33 ± 0.30	15.31 ± 0.60^a^	10.11 ± 0.57^c^	14.76 ± 1.29^b,d^
Creatinine (µmol/L)	84.14 ± 3.51	114.5 ± 21.40	70.28 ± 6.39	94.16 ± 7.0

Evaluation of lipid dysfunction for non-HDL. a: C vs D (p = 0.0106); b: C vs DI (p = 0.0210); c: D vs DQV(p = 0.0449). HDL fraction. a: C vs D (p < 0.0001); b: C vs DQV (p < 0.0001); c: C vs DI (p < 0.0001); d: D vs DQV (p = 0.0031). Total cholesterol (p = 0.0969). Triacylglycerol. a: C vs D (p = 0.0014); b: C vs DI (p < 0.0001); c: DQV vs DI (p < 0.0001). Hepatic function for ALT. a: C vs D (p < 0.0001); b: C vs DQV (p < 0.0001); c: C vs DI (p < 0.0001). AST. a: C vs D (p < 0.0001); b: C vs DQV (p = 0.0002); c: C vs DI (p < 0.0001); d: D vs DI (p = 0.0037); e: DQV vs DI (p = 0.0001). Renal function for albumin. a: C vs D (p < 0.0001); b: C vs DQV (p < 0.0001); c: C vs DI (p = 0.0010). Urea. a: C vs D (p < 0.0001); b: C vs DI (p < 0.0001); c: D vs DQV (p < 0.0001); d: DQV vs DI (p = 0.0005). Creatinine (p = 0.1125). C, control group (n = 6); D, diabetic group (n = 10); DQV, diabetic group treated with QV formulation (n = 14); DI, diabetic group treated with insulin (n = 10). Results expressed as mean ± standard error.

## Discussion

Glucose fluctuation is one of the main risk factors for patients with T1DM. As observed in this research, some prior results reported that DPP-4 inhibitors and Quercetin may reduce glycemic variations in the diabetes model^12,13^, however clinical trials and other research in T1DM indicate that administration of each of these agents separately is not consistent in altering glycemic parameters^8,14^, that is, results are conflicting and the final outcomes are inconclusive. The development of the QV formulation was mainly based on previous studies that showed that treatment with Vildagliptin and Quercetin alone did not increase serum insulin levels sufficiently to the extent of reducing glycemia (see Supplementary Table S1). Thus, in this study it was seen that the association of both agents (Vildagliptin and Quercetin) in a formulation was able to improve the hyperglycemic condition and promote a 70% reduction in hyperglycemia in relation to the 54.5% improvement achieved by treatment with insulin. Probably the improvement in glucose homeostasis contributed to a higher survival rate in animals treated with the QV formulation than untreated diabetic rats and diabetic rats treated with a daily dose of insulin, suggesting that the treatment with formulation may be associated with a low risk of the hypoglycemia that sometimes results from insulin administration^15^.

Glucose homeostasis is directly related to the hormonal balance of insulin and glucagon levels secreted by pancreatic cells. While the inhibitors of DPP-4 regulate glycemia by stimulating the release of insulin by pancreatic beta cells in a glucose-dependent manner, the role of Quercetin in glycemic control occurs mainly due to its antioxidant effects^10,11^. These inhibitors, by increasing the half-life of GLP-1, increase insulin synthesis and secretion, as well as reducing glucagon secretion by pancreatic alpha cells. Treatment of T1DM rats with Vildagliptin increased insulin secretion, however this increase was not sufficient to promote the lowering of blood glucose^7,8^. Some findings also revealed that Vildagliptin reduced glucagon during hyperglycemia and maintained its counterregulation during hypoglycemia in T1DM^16,17^, resulting in reduction in fasting plasma glucose, postprandial plasma glucose, and prevention of hypoglycemia. Glucagon, besides antagonizing the action of insulin, also induces gluconeogenesis and inappropriately raised glucagon secretion in T1DM patients contributed directly to increased hepatic glucose output and worsening of postprandial glucose control as well as variability of glycemia^18^. In our results, the treatment with the QV formulation was able to increase insulin levels, without altered glucagon levels. GLP-1R are scarce or nonexistent in pancreatic alpha cells, what makes it unlikely that GLP-1 has a direct action in reducing glucagon secretion in this cells^19^. Based on this finding, we can infer, to some extent, that glucose homeostasis is regulated primarily by insulin and to a lesser extent by glucagon. In addition, our hypothesis is supported since the insulin/glucagon ratio proved to be positive after the treatments, contributing to ameliorate the hyperglycemic state while it also maintains normal glucagon levels, and protects from hypoglycemia.

Glucose metabolism is influenced by a complex regulatory network that involves pancreatic and hepatic hexokinase enzyme activity. These enzymes are responsible for the phosphorylation of glucose and its entrapment in the intracellular environment. In pancreatic beta cells, hexokinase acts as a glucose sensor that activates insulin secretion. In hepatocytes, hexokinase activity is dependent on the insulin/glucagon ratio for the removal of excess glucose from the blood and its targeting to the glycolytic or glycogenesis pathway, through stimulation of glucose-induced glycogen synthesis^20,21^. In the absence of insulin, as occurs in individuals T1DM, hexokinase activity is reduced. Alloxan is a substance used as diabetogenic agent to induce T1DM in experimental animals and promotes selective inhibition of glucose-induced insulin secretion by inhibiting hexokinase^22^. This corroborates our findings regarding the inhibition of hexokinase activity in the group of untreated diabetic animals. Our results showed that, by preserving the pancreatic enzyme activity, the QV formulation increased insulin secretion and activated the liver enzyme to store glycogen in the hepatocytes, even after induction with alloxan. This effect probably occurred due to the action of Vildagliptin, which inhibits the DPP-4 enzyme and increases GLP-1 half-life, triggering exocytosis of insulin granules^23^ by activation of hexokinase after binding to its receptor. The GLP-1R are expressed in tissues other than pancreatic islets^24^, as in hepatocytes, but studies suggest that certain actions of GLP-1 in hepatic glucose metabolism occur through mechanisms that are independent of GLP-1R, and no alternative route seems to have been fully elucidated^25^. Administration of the QV formulation increased the pancreatic GLP-1R level, indicating a possible increase in the availability of GLP-1, whereas in the liver these levels did not change. Based on this, our results suggest that the sequence of modulatory effects on glucose metabolic homeostasis exhibited by the binding of GLP-1 to its receptor are possibly influenced, to a greater extent, by the interaction of GLP-1 with its pancreatic receptor and are less dependent on interaction with hepatic GLP-1R. Still regarding the signaling cascade, it should be noted that incretin-based treatment is able to increase the regulation of the GLUT2^22,26^. Vildagliptin is capable of preserving the expression of this transporter in the membrane of pancreatic beta cells^27^, while Quercetin reduces glucose absorption by inhibiting GLUT2 in intestinal cells^28^. However, even though alloxan did not inhibit the GLUT2 transporter function^29^, in this study, no alteration in GLUT2 levels was seen after administration of the QV formulation.

The QV formulation also had a positive effect on the lipid profile of diabetic rats, reducing non-HDL fraction and increasing HDL fraction, which is of particular significance because lipidic metabolism is directly related to dyslipidemia as well as microvascular and cardiovascular complications are typical of diabetes^30^. Atherosclerosis is a pathological condition resulting from lipid deposition and atheromatous plaque formation in the subendothelial layer of blood vessels, and oxidative stress and inflammatory responses are among the major triggers^31^. A study with patients with uncontrolled T2DM showed that Vildagliptin as an add-on to insulin therapy did not alter the lipid profile^32^, while Vildagliptin treatment ameliorated this parameter in diabetic mice^33^. The QV formulation had a beneficial effect in reducing atherogenic cholesterol fractions (decrease in non-HDL fraction accompanied by increase HDL fraction), while daily administration of insulin was responsible for increasing triacylglycerol levels when compared to treatment with the QV formulation. Although we did not observe improvement in triacylglycerol levels, some authors report that increased incretin plasma concentration is related to the reduction of intestinal absorption of triacylglycerol in animal studies^34^, besides Vildagliptin be able to lowers triacylglycerol-rich lipoprotein postprandial levels in T2DM, suggesting that DPP-4 inhibitors mobilize and burn fat during meals, and thus the fat that is associated with cardiovascular risk is not accumulated^35^. Our research group has previously suggested that treatment with Vildagliptin for 30 days improved the lipid profile in chronic T1DM model. Treatment with Vildagliptin alone reduced total cholesterol levels, and this reduction was due to decrease in the concentration of atherogenic fractions, since there was no change in the HDL fraction. Additionally, there was reduction of triacylglycerol levels^7^. Nath *et al*. also showed that Vildagliptin ameliorated lipid profile in diabetic mice^33^. In contrast, Maeda *et al*. did not observe alterations in total cholesterol, HDL fraction, and triacylglycerol levels in T1DM rats after treatment with Vildagliptin^36^. The effect of Quercetin has also been reported in the prevention and treatment of chronic non-infectious diseases, such as diabetes, obesity, and hyperlipidemia, mainly for its antioxidant effects. Studies have shown that Quercetin was able to improve the regulation of lipid metabolism^37^ and suppress the formation of atheromatous plaques in an atherosclerosis mouse model^31^. Therefore, supported by literature, our results reveal that 30-day treatment with QV formulation was effective in improving the lipid profile of animals.

Diabetes is responsible for causing damage to various organs including the liver and kidney. Assessment of liver function parameters revealed that QV formulation treatment did not reduce serum levels of ALT and AST enzymes. In contrast, treatment with insulin increased AST levels and since this enzyme is found in other compartments such as the heart, muscles, kidneys, and the brain and is released into the blood after some type of damage, it suggests the presence of some extrahepatic injury. Differently, the ALT enzyme is found largely in the liver, being released into the circulation as a result of liver damage. Alloxan hepatic metabolism may have been responsible for the increase in ALT/AST levels in diabetic animals, since the structural similarity with the glucose molecule allows its absorption by the cell through the GLUT2 transporter^38^, occasioning possible damage to cells which express this carrier. Treatment with the QV formulation also did not normalize the serum levels of albumin, a protein produced in the liver. Diabetes is responsible for reducing blood levels of albumin, which is also indicative of liver failure. Vildagliptin add-on insulin therapy in T2DM also did not alter the level of liver enzymes^32^, whereas in a hepatic fibrosis model the administration of Quercetin resulted in maintenance of ALT/AST levels and increased serum albumin levels, suggesting a possible hepatoprotective effect^39^. Diabetes is characterized by redox imbalance in the kidneys that can lead to nephropathy^40^. In addition, alloxan toxicity may also affect this organ due to the presence of the GLUT2 transporter in renal tubular cells^38^. Based on this, in relation to renal function, urea levels increased in diabetic animals, which may be justified by the higher protein catabolism and higher excretion of this metabolite. The QV formulation treatment, unlike the insulin treatment, promoted an improvement in this parameter to values similar to the control group, characterizing a possible renoprotective effect. Some studies indicate possible beneficial effects of DPP-4 inhibitors on renal complications caused by diabetes. In addition to providing protective effects against tubular damage and slowing the progression of chronic kidney disease, it has been suggested that the renoprotective effects of DPP-4 inhibitors may be due to increased levels of GLP-1^41^. Although our study showed no difference in creatinine levels, research using an experimental model of diabetic nephropathy showed that Quercetin treatment reduced serum creatinine, restored clearance, and reduced glomerular hyperplasia and oxidative stress^40^.

Metalloproteinases are extracellular matrix remodeling enzymes, and any change in their regulatory mechanism may contribute to complications in T1DM^42^. In this study, both the QV formulation and insulin reduced the MMP-2 activity, which was increased in untreated diabetic animals. Besides, we also confirmed that there is a strongly positive correlation between glycemic values and MMP-2 activity (see Supplementary Fig. S1). The hyperglycemic state, characteristic of T1DM, is responsible for the increase of MMP-2^43^. Peeters *et al*. in recent studies also associated higher MMP-2 plasma levels with higher incidence of cardiovascular events and diabetic nephropathy in T1DM patients^42^. Thus, the improvement in glycemic levels after the pharmacological treatments may justify the reduction in the activity of this enzyme.

Evaluation of pancreatic histology revealed that QV formulation was able to increase not only the number of pancreatic islets, but also the stored insulin content in the beta cells. These results allow us to infer that the QV formulation exerted a protective effect on the pancreatic tissue even after the alloxan induction. Progressive damage in beta cell function is the primary basis of diabetic manifestations^44^. In this sense, several authors have already reported a positive influence of DPP-4 inhibitors and GLP-1 on pancreatic tissue in the T2DM model, either by proliferation/maintenance of functional beta cells, inhibition of apoptosis or pancreatic islet neogenesis^9,45^. The islets express DPP-4 enzyme and can produce GLP-1. Thus, it is possible that the improvement in islet function it also results from inhibiting the local DPP-4 enzyme while the pancreatic GLP-1 is preserved^46^. This class of drugs improves pancreatic islet dysfunction, however are restricted the studies that verified these same effects in T1DM model, whether in the presence or absence of detectable beta cell function^47^. The close relationship involving hyperglycemia-mediated oxidative damage in T1DM has suggested that agents that improve the glycemic index and/or oxidative stress may have beneficial effects in the treatment of diabetes. This is the first study that demonstrates the effects of a formulation containing a DPP-4 inhibitor together with a Flavonoid, both with antidiabetic properties, in promoting the improvement of metabolic homeostasis in a T1DM model.

**Figure 7:**
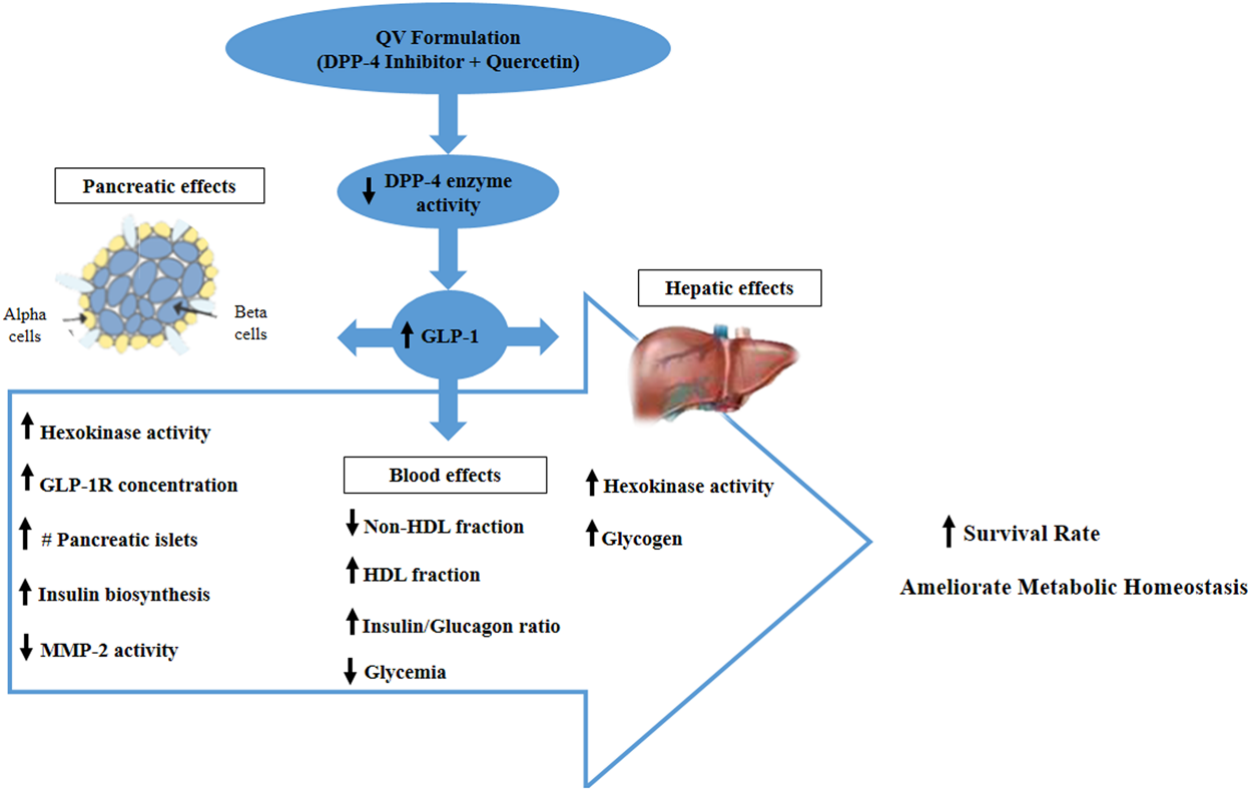
Proposed mechanism of action of QV formulation in the type 1 diabetes model. The QV formulation reduces the activity of the DPP-4 enzyme and promotes higher bioavailability of GLP-1, which is capable of promoting beneficial effects on blood parameters and on pancreatic and hepatic tissues. Together, these effects enable a higher survival rate and improvement in the metabolic homeostasis in the type 1 diabetes model. (↑) Increased, (↓) Decreased.

Thus, the present findings suggest a potential benefit of formulation QV in improves of metabolic homeostasis in rats (Fig. 7).

## Limitations

Alloxan is used to induce the characteristic symptoms of type 1 diabetes, however, should be emphasized that the etiogenesis of type 1 diabetes in an animal model is distinct from autoimmune type 1 diabetes observed in humans. The effects observed in this study with the treatment of diabetic animals with the QV formulation may be different from that observed in autoimmune diabetes. Therefore, future studies should be conducted to evaluate the effect of QV formulation in the treatment of autoimmune diabetes.

## Methods

### Chemicals

Vildagliptin (Novartis Pharma Stein AG, Stein, Switzerland) was commercially acquired in the pharmacy (Ouro Preto, Brazil). Quercetin and Alloxan (2, 4, 5, 6-tetraoxypyrimidine; 5, 6-dioxyuracil) was purchased from Sigma-Aldrich (St. Louis, MO, USA).

### Animals and Experimental Design

**Figure 8:**
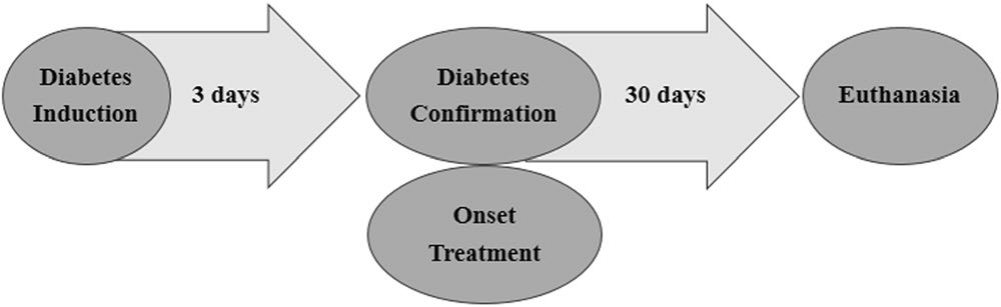
Timeline for the treatment of animals.

Forty-three female albino Fischer healthy rats (Laboratory of Experimental Nutrition of the University Federal of Ouro Preto), approximately 120 days of age and weighing 200 g, were used for this study. During the experimental period, the rats were maintained in well-ventilated cages with controlled temperature, ventilation, and humidity. They had access to water and commercial rat chow *ad libitum*. The animals were randomly divided into four experimental groups: untreated control animals (C) received 1 mL of the formulation containing the vehicle (n = 6); untreated diabetic animals (D) received 1 mL of the formulation containing the vehicle (n = 11); diabetic animals treated with QV formulation (DQV) received 1 mL of the formulation containing Vildagliptin^8,48,49^ (10 mg kg body mass)^−1^ and Quercetin (50 mg kg body mass)^−1^ (n = 14); and the diabetic animals treated with insulin (DI) received 2UI of insulin intraperitoneal injection* (n = 12). The animals of C, D, DQV and DI groups were submitted to orogastric treatment (oral gavage) daily, and in this order, for 30 consecutive days (Fig. 8). Animals were anesthetized by isoflurane inhalation and then euthanized. This work was conducted in accordance with the international standards of animal protection and the ethical principles of the National Council of Animal Experimentation and was approved by the Ethics Committee on Animal Use of the University Federal of Ouro Preto (#2014/17).

*Daily and for 30 consecutive days, at 9:00 AM, 2UI of exogenous insulin (NPH recombinant human insulin −10 mL suspension/100UI/mL concentration) were administered intraperitoneally and with the aid of a syringe. The insulin flask was kept under refrigeration (2–8 °C).

### Induction of Type 1 Diabetes

‘T1DM was induced by a single intraperitoneal injection of 135 mg alloxan (kg body mass)^−1^ dissolved in 0.2 mL sterile saline^22^. Control animals received only 0.2 mL of sterile saline solution. Three days after alloxan administration, animals were considered to be diabetic when after-fasting tail-vein blood glucose concentrations of ≥16 mmol/L, measured by an AccuChek Glucometer (Accu-Chek® Active, Roche).

### Biochemical Parameters

For biochemical analysis, the blood samples collected at the end of the experiment were used to measure glucose, triacylglycerol, total cholesterol, and high-density lipoprotein-cholesterol (HDL), alanine aminotransferase (ALT), aspartate aminotransferase (AST), urea and albumin levels all by Labtest Kits (Lagoa Santa, MG, Brazil). The serum insulin and glucagon levels were determined using a commercial Rat/Mouse Insulin enzyme-linked immunosorbent assay (ELISA) kit Merck Millipore (Kenilworth, NJ, USA) and by competitive inhibition enzyme immunoassay technique for quantitative measurement, using the enzyme-linked immunosorbent assay kit from Cloud-Clone Corp. (Katy, TX, USA), respectively.

The pancreas and liver were also collected at the end of the experiment. For determination of Hexokinase activity in pancreas and liver, 10 mg of tissues were rapidly homogenize with 200 µl ice cold HK Assay Buffer* for 10 minutes on ice. The homogenates were centrifuged at 12.000 rpm for 5 minutes e the supernatant was collect and store at −80 °C for further analysis. (Hexokinase Colorimetric Assay Kit – BioVision/Milpitas, CA, USA).

*HK Assay Buffer: Hexokinase Colorimetric Assay Kit (Catalog # K789-100).

For determination of GLUT2 (Glucose transporter 2) and GLP-1R (glucagon-like peptide 1 receptor) concentrations in pancreas and liver, tissues were rinsed in ice-cold PBS (0.01 mol.L^−1^, pH 7.0–7.2) to remove excess blood thoroughly and weighed before homogenization. Minced the tissues to small pieces (20 mg) and homogenized them in 1 mL lysis buffer* with a homogenizer on ice. The resulting suspension was sonicated with an ultrasonic cell disrupter to further break the cell membranes. After that, the homogenate was centrifuged for 5 minutes at 5.000 × g, for GLP-1R, and centrifuged for 5 minutes at 10.000 × g, for GLUT2. The respective supernatants were collected in aliquot and store at −80 °C for further analysis. Evaluated by sandwich enzyme immunoassay, using the Enzyme-linked Immunosorbent Assay Kit from Cloud-Clone Corp. (Katy, TX, USA).

*Lysis buffer: 50 mM.L^−1^ Tris-HCl pH 8,0; 1 mM.L^−1^ EDTA; 1% NP40; 150 mM.L^−1^ NaCl; 4 mL DTT.sample^−1^; 10 mL protease inhibitor/sample.

### Histological Parameters

For histological evaluation, the pancreas and liver fragments were fixed in 10% formaldehyde solution, dehydrated, diaphanized, and embedded in paraffin. The pancreas slides were stained with Hematoxylin and Eosin (H&E) to differentiate the architecture of pancreatic islets, and stained with anti-insulin antibody (1:5000 dilution) (Monoclonal Anti-Insulin, I2018-2mL, lot:084M4769, Sigma-Aldrich) for immunohistochemical staining of insulin in pancreatic tissue. (NOTE: Antibody Validation: positive and negative control of histological section of pancreatic islet immunolabeled with anti-insulin antibody). To quantify glycogen, the liver slides were stained with Periodic Schiff Acid (PAS). Photomicrographs were obtained using Leica Application Suite analysis software, magnification x440, and quantification was performed on Leica QWin Plus software version 3.0 (Leica Microsystems Inc., Buffalo Grove, IL, USA).

### Gelatin Zymography

MMP-2 activity was detected using gelatin zymography, as previously described by Sung *et al*.^50^.

### Statistical Analysis

The normality of the data was tested by the Kolmogorov-Smirnov test and followed normal distribution (parametric data). The results were expressed as mean ± standard error and statistical analysis was performed using One-Way Analysis of Variance (ANOVA), followed by Bonferroni post-test for multiple comparisons. Differences were considered statistically significant when p < 0.05. All analyses were performed using GraphPad Prism software version 6.01 for Windows (San Diego, California, USA).

### Ethics approval

All procedures followed were in accordance with the international standards of animal protection and the ethical principles of the National Council of Animal Experimentation and was approved by the Ethics Committee on Animal Use of the University Federal of Ouro Preto (#2014/17).

## Electronic supplementary material

Supplementary Information

## Data Availability

The datasets generated during and/or analysed during the current study are available from the corresponding author on reasonable request.
