# Supplementary material for: Oral formulation of DPP-4 inhibitor plus Quercetin improves metabolic homeostasis in type 1 diabetic rats
Source: Sci Rep. 2018 Oct 17;8:15310. doi: 10.1038/s41598-018-33727-x (PMC6192983; doi:10.1038/s41598-018-33727-x)
Supplement: Supplementary file 1 — Supplementary Information [file 41598_2018_33727_MOESM1_ESM.pdf]

Oral formulation of DPP-4 inhibitor plus Quercetin improves metabolic homeostasis in type 1 diabetic rats

Pedro Henrique de A. Miranda[a,b], Kissyla D. Lacerda[b,d], Carolina M. Araújo[b,e], José M. Barichello[e,f], Wanderson Lima[b,d], Daniela C. Costa[a,b,c]

[a]Laboratório de Bioquímica Metabólica (LBM), Departamento de Ciências Biológicas (DECBI), Universidade Federal de Ouro Preto (UFOP), Ouro Preto, MG 35400-000, Brazil.

[b]Programa de Pós-Graduação em Ciências Biológicas, Universidade Federal de Ouro Preto (UFOP), Ouro Preto, MG 35400-000, Brazil.

[c]Programa de Pós-Graduação em Saúde e Nutrição, Universidade Federal de Ouro Preto (UFOP), Ouro Preto, MG 35400-000, Brazil.

[d]Laboratório de Morfopatologia, Departamento de Ciências Biológicas (DECBI), Universidade Federal de Ouro Preto (UFOP), Ouro Preto, MG 35400-000, Brazil.

[e]Escola de Farmácia, Universidade Federal de Ouro Preto (UFOP), Ouro Preto, MG 35400-000, Brazil.

[f]Laboratório de Tecnologia Farmacêutica, Centro de Ciências Químicas, Farmacêutica e de Alimento, Universidade Federal de Pelotas (UFPEL), Rio Grande do Sul, RS 96160-990, Brazil

Experimental Groups	C	D	DQ	DV
**Final Glycemia (mmol/L)**	8.10 ± 0.61[a]	28.4 ± 0.90[b]	27.7 ± 0.57[b]	26.7 ± 1.63[b]
**Insulin (pmol/L)**	9.12 ± 0.70[a]	1.5 ± 0.47[b]	2.2 ± 0.12[b]	3.6 ± 0.70[b]

Supplementary Table S1. Evaluation of final glycemia and serum insulin of animals treated with Quercetin or Vildagliptin. Results expressed as mean ± standard error. Differences analysed by one-way ANOVA with Bonferroni multiple comparison test. Different letters indicate statistical difference when $p < 0.05$ ($p < 0.0001$ for Final Glycemia data and $p < 0.0001$ for Insulin dates). C, control group untreated (n=8), D, diabetic group untreated (n=7); DQ, diabetic group treated with Quercetin (5mg kg body mass)$^{-1}$ (n=8); DV, diabetic group treated with Vildagliptin (5mg kg body mass)$^{-1}$ (n=5).

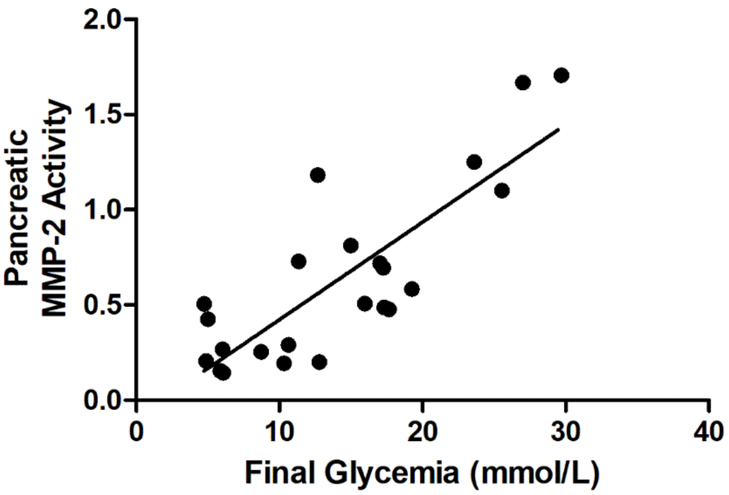

Supplementary Figure S1. Scatter plot illustrating the correlation between final glycemia and pancreatic MMP-2 activity. Strongly positive correlation between final glycemia and pancreatic MMP-2 activity. Pearson correlation (r = 0.8240) and correlation significant (alpha = 0.05) with value $p < 0.0001$.
